# A latent profile analysis of father support breastfeeding self-efficacy in the early postpartum period: a cross-sectional study

**DOI:** 10.3389/fnut.2026.1951173

**Published:** 2026-10-27

**Authors:** Junying Li, Qing Wang, Nuannuan Pan, Lan Zhang, Nafei Guo, Ying Liu

**Affiliations:** 1 Department of Obstetrics, Shanghai Key Laboratory of Maternal Fetal Medicine, Shanghai Institute of Maternal Fetal Medicine and Gynecologic Oncology, Shanghai First Maternity and Infant Hospital, School of Medicine, Tongji University, Shanghai, China; 2 Department of Nursing, Shanghai Key Laboratory of Maternal Fetal Medicine, Shanghai Institute of Maternal Fetal Medicine and Gynecologic Oncology, Shanghai First Maternity and Infant Hospital, School of Medicine, Tongji University, Shanghai, China; 3 Research Ward, Shanghai Key Laboratory of Maternal Fetal Medicine, Shanghai Institute of Maternal Fetal Medicine and Gynecologic Oncology, Shanghai First Maternity and Infant Hospital, School of Medicine, Tongji University, Shanghai, China

**Keywords:** breastfeeding, fathers, latent profile analysis, nutritional epidemiology, paternal breastfeeding support self-efficacy, postpartum period

## Abstract

**Background:**

Paternal support is a key determinant of breastfeeding success, with paternal breastfeeding support self-efficacy serving as the core cognitive foundation for fathers’ involvement in assisting their partners. However, existing research has focused on group-average levels, overlooking heterogeneity. This study used latent profile analysis (LPA) to identify subgroups of paternal breastfeeding support self-efficacy in the early postpartum period and to examine factors associated with profile membership.

**Methods:**

During September 2024 to February 2025, fathers of newborns were recruited by convenience sampling from a tertiary obstetrics and gynecology hospital in Shanghai. Measures included a general information questionnaire, a breastfeeding knowledge questionnaire, and the Father Support Breastfeeding Self-Efficacy Scale-Short Form (FBSES-SF). Latent profile models were estimated in Mplus 8.3, and factors associated with profile membership were examined using multivariable multinomial logistic regression via the three-step (R3STEP) procedure, which accounts for latent-class classification error.

**Results:**

The 306 fathers (mean age 31.69 ± 3.93 years) had a mean FBSES-SF total score of (53.03 ± 10.00). Three profiles emerged: low self-efficacy group (18.6%, *n* = 57), moderate self-efficacy group (64.7%, *n* = 198), and high self-efficacy group (16.7%, *n* = 51), which were largely parallel and best interpreted as severity gradations. Lower breastfeeding knowledge (OR = 0.90 per point, 95% CI 0.82–0.99) and perceiving breastfeeding as not important (OR = 9.44, 95% CI 1.65–53.91) were associated with membership in the low rather than the moderate group; perceiving breastfeeding as generally rather than very important (OR = 0.18, 95% CI 0.08–0.42) and expecting non-exclusive feeding (OR = 0.38, 95% CI 0.16–0.89) were associated with lower odds of membership in the high group. Associations were unchanged after adjustment for paternal age, education, and household income.

**Conclusion:**

Paternal breastfeeding support self-efficacy comprises three profiles that may guide early identification of fathers needing additional support. These findings may inform the development of stratified support strategies; whether such strategies improve paternal support behaviors or breastfeeding outcomes remains a hypothesis to be tested in future longitudinal and interventional studies.

## Introduction

Breast milk is globally recognized as the optimal nutritional source for infants, providing nearly all essential nutrients required for growth and development during the neonatal period and early infancy (1, 2). Beyond its nutritional value, breastfeeding confers significant health benefits for both mothers and infants. Breastfeeding not only reduces maternal risks of postpartum depression, breast cancer, ovarian cancer, osteoporosis, cardiovascular diseases, and diabetes (3–5), but also enhances maternal and infant mental health and emotional bonding (6), while lowering the risks of neonatal infectious diseases, constipation, and childhood obesity (7, 8). Furthermore, longer breastfeeding duration has been linked to higher intelligence quotients and improved academic performance in later childhood (9, 10). The World Health Organization (WHO) explicitly recommends exclusive breastfeeding for infants within the first 6 months and continued breastfeeding until at least 2 years of age. China’s National Health Commission has issued the “Action Plan for Promoting Breastfeeding (2021–2025),” aiming to achieve an exclusive breastfeeding rate exceeding 50% among infants under 6 months by June 2025. However, according to China’s national Under-5 Child Nutrition and Health Surveillance System (U5CNHSS), the prevalence of exclusive breastfeeding (defined as feeding infants under 6 months of age with breast milk only, without any liquid foods or solid/semi-solid/soft foods during the previous 24 h) was 34.9% (11), which remains below the global average (48%) (12).

The success of breastfeeding is influenced by multiple factors, among which paternal support plays a critical role in maternal decisions to initiate and sustain breastfeeding, as well as in maternal breastfeeding self-efficacy (13–15). According to Bandura’s Social Cognitive Theory (16), individual self-efficacy not only directly affects behavior initiation and persistence but also influences decision-making through mechanisms such as observational learning, verbal encouragement, and emotional support. Paternal breastfeeding support self-efficacy—defined as a father’s confidence in his ability to assist his partner in breastfeeding—constitutes the core cognitive foundation underpinning effective paternal involvement (17), and has been identified as a key moderating factor in infant feeding practices (18). Fathers with higher breastfeeding self-efficacy demonstrate greater confidence and competence in engaging with the breastfeeding process; such active involvement, in turn, encourages more frequent provision of emotional and practical support, thereby increasing the likelihood of successful breastfeeding (19). In contrast, fathers with low self-efficacy, although often willing to support breastfeeding, frequently lack the confidence or preparedness to offer effective assistance, which may inadvertently undermine their partner’s breastfeeding efforts (20, 21).

Within the Social Cognitive Theory, the variables examined in the study can be classified into three conceptually distinct groups. First, sociodemographic characteristics and prior attendance at breastfeeding education sessions represent personal and experiential antecedents that correspond to sources of efficacy information (e.g., vicarious learning and verbal persuasion). Second, concurrent cognitive and contextual correlates measured at the same time as self-efficacy (including breastfeeding knowledge, perceived importance of breastfeeding, expected feeding method, and the father-perceived maternal lactation context);because the design is cross-sectional, the direction of these associations cannot be determined, and some of these variables (particularly current and expected feeding methods) could equally be consequences of self-efficacy rather than antecedents. Third, downstream consequences of self-efficacy, such as actual supportive behaviors and longer-term breastfeeding outcomes, were not measured in the present study.

Existing research on paternal support for breastfeeding predominantly provides overall descriptive analyses, focus on single influencing factors, or rely solely on total scale scores to assess paternal self-efficacy in breastfeeding support, inter-individual heterogeneity is frequently neglected (19, 22). Given this methodological limitation, reliance on mean scores tends to oversimplify complex phenomena and obscure subgroup differences: fathers with the same average score may differ considerably in their confidence profiles, and person-centered studies examining heterogeneity in paternal breastfeeding support self-efficacy are lacking. As a result, there is still insufficient clarity regarding the true baseline distribution of paternal breastfeeding support self-efficacy, the heterogeneity in their profiles and the key factors that shape these differences. In the absence of a robust “baseline map,” future interventions risk being poorly targeted, which may dilute their effects, complicate result interpretation, and waste primary healthcare resources. It also becomes challenging to judge whether current paternal involvement in breastfeeding support interventions has advanced faster than the evidentiary foundation needed to support them.

Latent Profile Analysis (LPA) is a person-centered analytical method that, through modeling of continuous variables, categorizes individuals into several relatively homogeneous groups based on distinct behavioral patterns, thereby revealing population heterogeneity (23). Recently, LPA has been widely applied in psychology, medicine, nursing management, and education (24). This method classifies individuals based on data-driven patterns, captures population heterogeneity and enables identification of distinct paternal profiles in breastfeeding support—a critical step in addressing competency gaps and developing targeted intervention strategies. Accordingly, this study uses LPA, focusing on infant fathers, to explore latent profiles of paternal breastfeeding support self-efficacy in the postpartum, describe the characteristics of each profile, and examine factors associated with profile membership.

## Methods

### Study design

This cross-sectional study was conducted in accordance with the STROBE guidelines.

### Study setting

This study was conducted from September 2024 to February 2025 in Shanghai, one of China’s largest cities and economic centers. The subjects were recruited from Shanghai First Maternity and Infant Hospital, School of Medicine, Tongji University, which is one of the earliest provincial and municipal maternal and child health care institutions in China. It has more than 800 beds and a maximum annual delivery capacity of 30,000 (Shanghai First Maternity and Infant Hospital, 2020). Fathers were approached and recruited in the postnatal wards during their partners’ hospitalization after childbirth, using convenience sampling. All fathers completed the survey in the postnatal ward before their partner’s discharge, predominantly on the second to third postpartum day; the early postpartum period in this study therefore refers to the first days after birth, preceding hospital discharge. Potential participants were informed of the study purpose and had the right to refuse or withdraw at any time without any consequence. After providing written informed consent (see Ethical approval and consent to participate), participants completed an anonymous electronic questionnaire.

### Study population

Eligible participants were fathers aged ≥22 years whose partners were ≥ 20 years (these thresholds correspond to the legal minimum marriage ages in mainland China), with a singleton birth and no maternal or neonatal contraindications to breastfeeding (Maternal contraindications included heart disease with New York Heart Association class III–IV cardiac function, AIDS, syphilis, or viral hepatitis; Neonatal contraindications included birth asphyxia or severe infection). Fathers were required to be able to read and understand Chinese and to complete the questionnaire independently, also they must voluntarily participate in this study and sign an informed consent form. Exclusion criteria included intellectual or psychiatric conditions affecting survey completion, or severe communication impairments.

### Sample

The sample size was determined based on methodological recommendations for latent profile analysis. Simulation studies indicate that, with a sufficient number of indicators, clear class separation, and no extremely small classes, stable latent profile solutions can be obtained with samples of approximately 300 or more (25–27). The final sample comprised 306 participants, meeting this recommendation: the profile model included 14 indicators, the entropy of the retained three-class solution was 0.977 (indicating clear class separation), and the smallest class comprised 16.7% of the sample (*n* = 51), indicating adequate classification precision.

### Data collection

The study received approval from the participating hospital. An electronic questionnaire was created using the online survey platform Questionnaire Star^1^, and a QR code was generated for distribution. The landing page outlined the study purpose, significance, instructions, and data confidentiality agreement. With the support of nursing managers, participants were informed about the study details and provided informed consent prior to questionnaire distribution. To ensure data quality, standardized explanations were provided in case of questions during completion; the survey system was configured to prompt respondents for any missing items and to restrict each IP address to a single submission. Two researchers independently reviewed all submissions using exclusion rules defined *a priori*, before any data analysis: (a) completion in less than two minutes—during pilot testing before the survey, five research team members and individuals who had never seen the questionnaire completed it in approximately 3–7 min, and even team members familiar with its content needed at least about 3 min, so responses completed in under two minutes were considered implausible as considered responses; or (b) illogical responses, defined as random or straight-line answering—endorsement of nearly the same option across all scaled items, indicating that the questionnaire had been filled in carelessly. Discrepancies between the two reviewers were resolved by discussion.

### Measurements

#### General information questionnaire

In this study, a self-designed questionnaire was used to collect general information: (a) demographic and obstetric characteristics: paternal and maternal age, education level, household per capita monthly income, occupation, census register, parity, pregnancy complications, mode of delivery, and neonatal gender; (b) the following study variables: All items were answered by the fathers themselves in the postnatal ward on the second to third postpartum day, before their partner’s discharge—including the maternal variables, which therefore reflect fathers’ perceptions rather than maternal self-report or clinical assessment. The exact wording and response categories were as follows: feeding methods before discharge (“Which feeding method is your partner currently using?”—exclusive breastfeeding) [breastfeeding only, without any supplements]/mixed feeding [breastfeeding supplemented with other infant formulas]/formula feeding [fed exclusively with infant formulas, etc.]; expected feeding method (“Which feeding method do you expect to use after discharge?”—exclusive breastfeeding) [breastfeeding only, without any supplements]/mixed feeding [breastfeeding supplemented with other infant formulas]/formula feeding [fed exclusively with infant formulas, etc.]; paternal perceived importance of breastfeeding (“Do you think breastfeeding is important?”—not important at all/not very important/generally important/ important/very important); prior attendance at breastfeeding education sessions (“Have you previously attended breastfeeding education courses together with your partner?”—yes/no); maternal lactation status (“How is your partner’s current milk supply?”—sufficient/insufficient/Not yet established); maternal breastfeeding fatigue (“How is your partner’s current fatigue with breastfeeding?”—no fatigue/mild fatigue/ moderate fatigue /severe fatigue); maternal feeding-related pain (“How much nipple pain does your partner have during breastfeeding?”—no pain/mild pain/moderate pain/severe pain).

#### Father Support Breastfeeding Self-Efficacy Scale-Short Form

The original questionnaire was developed in 2018 by Canadian researchers Dennis et al. (28), this scale is adapted from the original Breastfeeding Self-Efficacy Scale to assess fathers’ confidence in supporting breastfeeding. This unidimensional scale comprises 14 items, each rated on a 5-point Likert scale ranging from 1 (not at all confident) to 5 (very confident), with total scores from 14 to 70. Higher scores indicate greater paternal confidence in breastfeeding support. The Cronbach’s α coefficient for this scale ranges from 0.91 to 0.92. In 2020, Chinese researcher Zhou Yang (29) localized and calibrated the scale, obtaining a Cronbach’s α coefficient of 0.902–0.938.

Breastfeeding Knowledge Questionnaire: This 17 items questionnaire, originally developed by Zhao Min (30), assesses breastfeeding knowledge across two domains: the benefits of breastfeeding and breastfeeding skills; the original questionnaire had a content validity index (CVI) of 0.91. In this study, item wording was minimally adapted so that all items were answerable by fathers. Items are answered as yes/no/uncertain; each correct response is scored 1 point (incorrect or uncertain, 0), with total scores ranging from 0 to 17; higher scores indicate more comprehensive breastfeeding knowledge. The questionnaire has previously been administered to paternal populations in China with acceptable internal consistency (Cronbach’s α = 0.89 in a sample of 507 fathers of infants and toddlers) (31).

### Statistical analysis

Data were analyzed using SPSS 20.0 and Mplus 8.3 software. Categorical variables were summarized as frequencies; continuous variables were expressed as mean ± standard deviation or median (interquartile range). Group comparisons were conducted using independent *t*-tests, one-way ANOVA, or their non-parametric equivalents. Latent profile analysis was conducted in Mplus 8.3 using the 14 FBSES-SF items as continuous indicators, with the robust maximum likelihood estimator (MLR). Treating the 14 five-point items as continuous indicators follows standard practice in LPA, and simulation studies have shown that Likert-type items with five or more response categories can be analyzed as continuous without introducing meaningful bias (32). Consistent with the conditional (local) independence assumption, within-class covariances among indicators were fixed to zero. Item means were freely estimated across classes, while item variances were constrained to be equal across classes (58 free parameters for the three-class model), for parsimony and because freeing variances with skewed Likert-type indicators can extract spurious, distribution-driven classes (33). To avoid convergence to local maxima, 1,000 random starts with 250 final-stage optimizations were used, and the best log-likelihood value was confirmed to be replicated. Model selection was based on the Akaike information criterion (AIC), Bayesian information criterion (BIC), and sample-size adjusted BIC (aBIC), entropy, the Lo–Mendell–Rubin adjusted likelihood ratio test, and the parametric bootstrapped likelihood ratio test (500 bootstrap draws). Lower AIC/BIC/aBIC values indicate better fit; entropy values closer to 1 indicate higher classification accuracy; and a significant LMR-LRT or BLRT indicates that a k-class model fits better than the (k − 1)-class model. To examine whether the retained profiles differed only in overall level or also in shape, a Wald test of parameter constraints was used to test the hypothesis that the profiles were strictly parallel across the 14 items.

Factors associated with profile membership were examined using the three-step procedure (R3STEP) in Mplus 8.3, which regresses latent class membership on covariates while accounting for classification error (34, 35). Predictor selection was theory-informed rather than based on univariate screening: guided by the conceptual framework, eight prespecified predictors were entered simultaneously, namely breastfeeding knowledge, prior attendance at breastfeeding education sessions, paternal perceived importance of breastfeeding, maternal feeding-related pain, maternal breastfeeding fatigue, maternal lactation status, feeding method before discharge, and expected feeding method. As a sensitivity analysis, key demographic covariates (paternal age, educational attainment, and household per-capita monthly income) were additionally entered. All coefficients and dummy-category contrasts are reported regardless of statistical significance, together with odds ratios (ORs) and 95% confidence intervals (CIs), model likelihood-ratio statistics, pseudo-*R*^2^ values, and the sample size of each analysis. Two-sided *p* < 0.05 was considered statistically significant.

## Results

### Participant characteristics

A total of 355 questionnaires were distributed in this study, of which 49 were excluded according to the *a priori* rules (32 completed in under two minutes and 17 showing random or straight-line answering), leaving 306 valid responses (valid response rate 86.2%). Participants had a mean age of 31.69 years (SD = 3.93; range: 24–47), and their partners had a mean age of 30.46 years (SD = 3.54; range: 22–42). Over half of the participants (58.5%) were migrant from other regions; 71.6% held a bachelor’s degree or higher; and 47.4% reported a monthly household income ranging between 10,000 and 20,000 yuan. More than half of the fathers (51.3%) expressed a preference for exclusive breastfeeding, and the vast majority (95.5%) considered breastfeeding to be important; however, only 44.8% had attended antenatal breastfeeding education sessions with their partners. Detailed characteristics by profile are presented in Table 1.

**Table 1:** Comparison of socio-demographic characteristics of fathers with different levels of self-efficacy in supporting breastfeeding (percentages, %).

Variables	Classification	Number (%, *n* = 306)	Low self-efficacy [57 (18.6%)]	Moderate self-efficacy [198 (64.7%)]	High self-efficacy [51 (16.7%)]	χ^2^/*H*	*P*
Maternal age	≤25	19 (6.2)	0 (0.0)	16 (8.1)	3 (5.9)	5.310^b^	0.257
	25–35	263 (85.9)	52 (91.2)	166 (83.8)	45 (88.2)		
	>35	24 (7.8)	5 (8.8)	16 (8.1)	3 (5.9)		
Paternal age	≤25	11 (3.6)	0 (0.0)	9 (4.5)	2 (3.9)	5.825^b^	0.213
	25–35	251 (82.0)	46 (80.7)	166 (83.8)	39 (76.5)		
	>35	44 (14.4)	11 (19.3)	23 (11.6)	10 (19.6)		
Education level	Specialized degree or lower	87 (28.4)	16 (28.1)	54 (27.3)	17 (33.3)	2.064^a^	0.724
	Undergraduate degree	150 (49.0)	25 (43.9)	101 (51.0)	24 (47.1)		
	Master’s degree or higher	69 (22.6)	16 (28.1)	43 (21.7)	10 (19.6)		
Occupation	Company employee	204 (66.7)	38 (66.7)	130 (65.7)	36 (70.6)	1.722^a^	0.787
	Other work	70 (22.9)	11 (19.3)	48 (24.2)	11 (21.6)		
	Liberal professions	32 (10.5)	8 (14.0)	20 (10.1)	4 (7.8)		
Household per capita monthly income (yuan)	<10,000	76 (24.8)	18 (31.6)	43 (21.7)	15 (29.4)	3.499^a^	0.478
	10,000–20,000	145 (47.4)	24 (42.1)	100 (50.5)	21 (41.2)		
	>20,000	85 (27.8)	15 (26.3)	55 (27.8)	15 (29.4)		
Census register	Shanghai	127 (41.5)	23 (40.4)	79 (39.9)	25 (49.0)	1.428^a^	0.490
	Other places	179 (58.5)	34 (59.6)	119 (60.1)	26 (51.0)		
Mode of delivery	Vaginal delivery (including operative vaginal deliveries)	171 (55.9)	28 (49.1)	113 (57.1)	30 (58.8)	1.349^a^	0.509
	Caesarean section	135 (44.1)	29 (50.9)	85 (42.9)	21 (41.2)		
Pregnancy complications	Yes	59 (19.3)	9 (15.8)	36 (18.2)	14 (27.5)	2.787^a^	0.248
	No	247 (80.7)	48 (84.2)	162 (81.8)	37 (72.5)		
Neonatal gender	Boy	160 (52.3)	26 (45.6)	100 (50.5)	34 (66.7)	5.496^a^	0.064
	Girl	146 (47.7)	31 (54.4)	98 (49.5)	17 (33.3)		
Parity	First birth	234 (76.5)	43 (75.4)	156 (78.8)	35 (68.6)	2.368^a^	0.306
	Second birth or more	72 (23.5)	14 (24.6)	42 (21.2)	16 (31.4)		
Expected feeding method	Exclusive breastfeeding	157 (51.3)	15 (26.3)	103 (52.0)	39 (76.5)	27.216^a^	<0.001
	Non-exclusive breastfeeding	149 (48.7)	42 (73.7)	94 (48.0)	11 (23.5)		
Feeding method before discharge	Exclusive breastfeeding	123 (40.2)	16 (28.1)	88 (44.4)	19 (37.3)	5.157^a^	0.076
	Non-exclusive breastfeeding	183 (59.8)	41 (71.9)	110 (55.6)	32 (62.7)		
Maternal lactation status	Not yet established	15 (4.9)	7 (12.3)	4 (2.0)	4 (7.8)	25.930^a^	<0.001
	Insufficient	154 (50.3)	37 (64.9)	101 (51.0)	16 (31.4)		
	Sufficient	137 (44.8)	13 (22.8)	93 (47.0)	31 (60.8)		
Maternal breastfeeding fatigue	No fatigue	45 (14.7)	4 (7.0)	31 (15.7)	10 (19.6)	9.798^a^	0.044
	Mild fatigue	160 (52.3)	25 (43.9)	108 (54.5)	27 (52.9)		
	Moderate to severe fatigue	101 (33.0)	28 (49.1)	59 (29.8)	14 (27.5)		
Maternal feeding-related pain	No or mild pain	185 (60.5)	26 (45.6)	125 (63.1)	34 (66.7)	6.668^a^	0.036
	Moderate to severe pain	121 (39.5)	31 (54.4)	73 (36.9)	17 (33.3)		
Paternal perceived importance of breastfeeding	Not important	14 (4.5)	8 (14.0)	4 (2.0)	2 (3.9)	56.979^b^	<0.001
	Generally important	170 (55.6)	39 (68.4)	122 (61.6)	9 (17.6)		
	Very important	122 (39.9)	10 (17.5)	72 (36.4)	40 (78.4)		
Prior attendance at breastfeeding education sessions	Yes	137 (44.8)	15 (26.3)	88 (44.4)	34 (66.7)	17.748^a^	<0.001
	No	169 (55.2)	42 (73.7)	110 (55.6)	17 (33.3)		
Breastfeeding knowledge ( $\bar{x}+s$ )			10.47 ± 4.36	12.53 ± 3.38	13.63 ± 2.94	18.501^c^	<0.001

a χ^2^ = chi-square tests.

b Monte Carlo exact test based on 100,000 random permutations, used when more than 20% of cells had expected counts below 5.

c H=Kruskal-Wallis H.

*p* < 0.05 was considered statistically significant.

### The scores of the main variables

The mean total FBSES-SF score was (53.03 ± 10.00), with a mean item score of (3.79 ± 0.71). The Cronbach’s α was 0.951. Because no validated cut-offs exist for the FBSES-SF, scores are reported descriptively without attaching a qualitative level. The mean total Breastfeeding Knowledge score was 12.33 ± 3.63, with a mean item score of 0.73 ± 0.21. The Cronbach’s α in the present paternal sample was 0.824.

### Latent profile analysis and naming of the profiles

Latent profile models with one to five classes were fitted using the 14 FBSES-SF items as indicators (Table 2). Although the AIC, BIC, and aBIC decreased as the number of classes increased, the LMR-LRT was non-significant for both the four-class (*p* = 0.070) and five-class (*p* = 0.514) solutions, indicating that extracting additional classes did not significantly improve model fit. Moreover, the four-class solution yielded a small class comprising only 5.9% of the sample, raising concerns about stability and interpretability, and its entropy was only marginally higher than that of the three-class model (0.980 vs. 0.977). The BLRT remained significant across all solutions; because this test tends to favor additional classes in practice, it was not used as the sole selection criterion (25). Considering statistical fit, parsimony, class size, and interpretability jointly, the three-class model was retained. A Wald test of parameter constraints rejected the hypothesis that the three profiles were strictly parallel across items (χ^2^ (26) = 107.983, *p* < 0.001), indicating that the classes differed in item-level patterns and not merely in overall level. This interpretation was supported by sensitivity analyses: a single-factor confirmatory factor analysis (CFA) fitted the data reasonably well (χ^2^(77) = 190.239, *p* < 0.001; RMSEA = 0.069, 90% CI: 0.057–0.082; CFI = 0.935; TLI = 0.924; BIC = 8482.9), and factor-mixture models combining one continuous factor with two or three latent classes did not improve on the CFA (BIC = 8488.7 and 8494.4, respectively; both solutions converged toward the factor-only solution, with no meaningful class separation beyond the general factor). Nevertheless, differences between adjacent classes ranged from 0.72 to 1.43 scale points across items, and the profiles were largely parallel in appearance (Figure 1), suggesting that they primarily represent severity gradations of the same underlying construct. The three profiles are shown in Figure 1. The mean FBSES-SF total scores for Classes 1, 2, and 3 were 37.04 (SD = 6.77), 53.99 (SD = 3.58), and 67.20 (SD = 3.08), respectively. Each profile was named based on its score characteristics: Profile 1, comprising 57 participants (18.6%) with the lowest mean scores, was labeled the low self-efficacy group; Profile 2, comprising 198 participants (64.7%) with moderate mean scores, was labeled the moderate self-efficacy group; and Profile 3, comprising 51 participants (16.7%) with the highest mean scores, was labeled the high self-efficacy group.

**Table 2:** Model fit results for latent profile analysis (*n* = 306).

Model	AIC	BIC	aBIC	Entropy	LMR	BLRT	Class probabilities
1	11355.437	11459.698	11370.895	–	–	–	–
2	9624.065	9784.180	9647.803	0.981	<0.001	<0.001	0.212/0.788
3	8724.789	8940.757	8756.808	0.977	0.0149	<0.001	0.186/0.647/0.167
4	8354.397	8626.219	8394.697	0.980	0.0704	<0.001	0.059/0.611/0.163/0.167
5	8224.654	8552.329	8273.234	0.957	0.5142	<0.001	0.059/0.140/0.137/0.507/0.157

**Figure 1:**
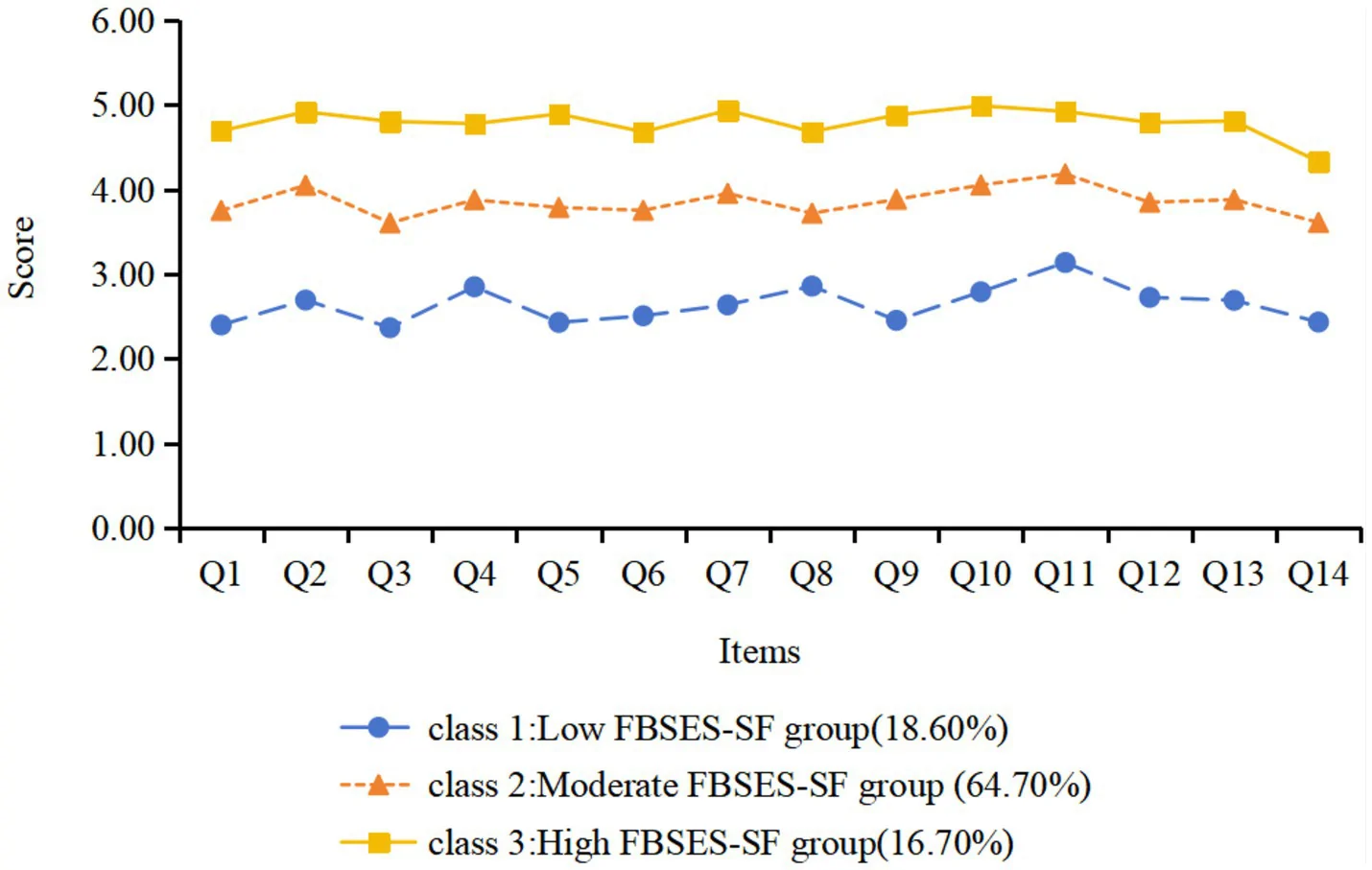
Distribution characteristics of latent profiles in paternal breastfeeding support self-efficacy in the early postpartum period.

### Univariate comparisons across latent profiles

The result of univariate analysis showed that the breastfeeding knowledge scores were (10.47 ± 4.36) in the “low self-efficacy group,” (12.53 ± 3.38) in the “moderate self-efficacy group,” and (13.63 ± 2.94) in the “high self-efficacy group.” Comparisons among the three groups revealed statistically significant differences (*H* = 18.501, *p* < 0.001). Also, the differences in the distribution of the latent profiles of the expected feeding method, maternal lactation status, maternal breastfeeding fatigue, maternal feeding-related pain, paternal perceived importance of breastfeeding and prior attendance at breastfeeding education sessions were statistically significant (*p* < 0.05). No significant differences were observed for other demographic variables (*p* > 0.05; Table 1), Further details are presented in Table 1. These comparisons are descriptive; all prespecified candidate predictors were entered into the multivariable model regardless of univariate significance.

### Multicollinearity assessment

To assess the suitability of the selected explanatory and control variables for regression analysis, variance inflation factors (VIF) and tolerance statistics were calculated to quantify multicollinearity issues. According to established methodological standards, VIF values between 0 and 5 indicate acceptable collinearity levels. In this study, the VIF values for all predictors (including the demographic covariates in the sensitivity model) ranged from 1.059 to 2.525 (Table 3), indicating no substantial multicollinearity among the independent variables.

**Table 3:** Variance inflation factors (VIF) and tolerance statistics for all predictors.

Variable (reference category)	VIF	Tolerance
Paternal age: ≤25 years (ref: 25–35 years)	1.073	0.932
Paternal age: >35 years (ref: 25–35 years)	1.059	0.944
Education level: master’s degree or higher (ref: undergraduate degree)	1.203	0.831
Education level: specialized degree or lower (ref: undergraduate degree)	1.241	0.806
Household per capita monthly income (yuan): <10,000 (ref: 10,000–20,000)	1.234	0.810
Household per capita monthly income (yuan): >20,000 (ref: 10,000–20,000)	1.222	0.818
Feeding method before discharge: non-exclusive breastfeeding (ref: exclusive breastfeeding)	1.166	0.858
Expected feeding method: non-exclusive breastfeeding (ref: exclusive breastfeeding)	1.197	0.835
Maternal lactation status: insufficient (ref: sufficient)	1.279	0.782
Maternal lactation status: not yet established (ref: sufficient)	1.356	0.738
Maternal breastfeeding fatigue: mild fatigue (ref: no fatigue)	2.318	0.431
Maternal breastfeeding fatigue: moderate-to-severe (ref: no fatigue)	2.525	0.396
Maternal feeding-related pain: moderate-to-severe pain (ref: no or mild pain)	1.224	0.817
Paternal perceived importance of breastfeeding: generally important (ref: very important)	1.235	0.810
Paternal perceived importance of breastfeeding: not important (ref: very important)	1.162	0.861
Prior attendance at breastfeeding education sessions: no (ref: yes)	1.130	0.885
Breastfeeding knowledge (continuous)	1.187	0.843

### Factors associated with latent profile membership

Factors associated with profile membership were examined using the three-step (R3STEP) procedure in Mplus 8.3, which accounts for classification error in latent class assignment (classification accuracy was very high: entropy = 0.977; average posterior probabilities of most likely class membership 0.98–0.99). The moderate self-efficacy group served as the reference outcome category; predictor coding and reference categories are shown in Table 4, and the complete set of estimates for both contrasts (low vs. moderate; high vs. moderate) is reported in Table 5 (main model) and Table 6 (sensitivity model).

**Table 4:** Coding of candidate predictors in the multinomial logistic regression (R3STEP).

Predictor	Coding (reference category)
Paternal age (sensitivity model only)	25–35 years = reference (0, 0); ≤25 years = (1, 0); >35 years = (0, 1)
Education level (sensitivity model only)	Undergraduate degree = reference (0, 0); master’s degree or higher = (1, 0); Specialized degree or lower = (0, 1)
Household per-capita monthly income (sensitivity model only)	10,000–20,000 yuan = reference (0, 0); <10,000 yuan = (1, 0); >20,000 yuan = (0, 1)
Feeding method before discharge	Exclusive breastfeeding = 0 (reference); Non-exclusive breastfeeding = 1
Expected feeding method	Exclusive breastfeeding = 0 (reference); Non-exclusive breastfeeding = 1
Maternal lactation status	Sufficient = reference (0, 0); insufficient = (1, 0); not yet established = (0, 1)
Maternal breastfeeding fatigue	No fatigue = reference (0, 0); mild fatigue (1,0); moderate to severe fatigue (0,1)
Maternal feeding-related pain	No or mild pain = 0 (reference); moderate to severe pain = 1
Paternal perceived importance of breastfeeding	Very important = reference (0, 0); generally important = (1, 0); not important = (0, 1)
Prior attendance at breastfeeding education sessions	Yes = 0 (reference); No = 1
Breastfeeding knowledge	Continuous total score (0–17)

**Table 5:** Factors associated with latent profile membership of paternal breastfeeding support self-efficacy: multivariable multinomial logistic regression using the three-step (R3STEP) procedure (*n* = 306).

Predictor (reference category)	Low self-efficacy group vs. Moderate self-efficacy group			High self-efficacy group vs. Moderate self-efficacy group		
	*β* (SE)	*P*	*OR* (95%*CI*)	*β* (SE)	*P*	*OR* (95%*CI*)
Breastfeeding knowledge (per one-point increase)	−0.104 (0.046)	0.024	0.90 (0.82–0.99)	0.075 (0.072)	0.302	1.08 (0.94–1.24)
Prior attendance at breastfeeding education sessions: no (ref: yes)	0.657 (0.401)	0.101	1.93 (0.88–4.23)	−0.596 (0.386)	0.122	0.55 (0.26–1.17)
Paternal perceived importance of breastfeeding: generally important (ref: very important)	0.591 (0.447)	0.186	1.81 (0.75–4.34)	−1.701 (0.422)	<0.001	0.18 (0.08–0.42)
Paternal perceived importance of breastfeeding: not important (ref: very important)	2.245 (0.889)	0.012	9.44 (1.65–53.91)	0.413 (0.876)	0.637	1.51 (0.27–8.41)
Maternal feeding-related pain: moderate-to-severe pain (ref: no or mild pain)	0.307 (0.410)	0.455	1.36 (0.61–3.04)	−0.279 (0.406)	0.493	0.76 (0.34–1.68)
Maternal breastfeeding fatigue: mild fatigue (ref: no fatigue)	0.994 (0.719)	0.167	2.70 (0.66–11.06)	−0.156 (0.498)	0.754	0.86 (0.32–2.27)
Maternal breastfeeding fatigue: moderate-to-severe fatigue (ref: no fatigue)	1.327 (0.746)	0.076	3.77 (0.87–16.27)	−0.279 (0.590)	0.637	0.76 (0.24–2.40)
Maternal lactation status: insufficient (ref: sufficient)	0.612 (0.396)	0.123	1.84 (0.85–4.01)	−0.600 (0.391)	0.125	0.55 (0.26–1.18)
Maternal lactation status: not yet established (ref: sufficient)	1.646 (0.876)	0.060	5.19 (0.93–28.87)	1.403 (0.966)	0.147	4.07 (0.61–27.01)
Feeding method before discharge: non-exclusive breastfeeding (ref: exclusive breastfeeding)	0.394 (0.384)	0.305	1.48 (0.70–3.15)	0.557 (0.391)	0.154	1.75 (0.81–3.76)
Expected feeding method: non-exclusive breastfeeding (ref: exclusive breastfeeding)	0.661 (0.402)	0.101	1.94 (0.88–4.26)	−0.963 (0.432)	0.026	0.38 (0.16–0.89)

Three-step (R3STEP) estimates from Mplus 8.3, which accounts for latent-class classification error (entropy = 0.977; average posterior probabilities of most likely class membership 0.98–0.99). Reference outcome category: moderate self-efficacy group (*n* = 198); low self-efficacy group *n* = 57; high self-efficacy group *n* = 51. Reference categories of predictors are shown in parentheses. Model likelihood-ratio χ^2^ (22) = 114.61, *p* < 0.001; McFadden *R*^2^ = 0.210; Cox–Snell *R*^2^ = 0.312; Nagelkerke *R*^2^ = 0.375; likelihood-ratio statistics and pseudo-*R*^2^ coefficients were computed from the log-likelihood values of the fitted three-step models. 95% CIs were computed from robust (MLR) standard errors. Sparse cells: paternal perceived importance “not important” (8, 4, and 2 fathers in the low, moderate, and high groups, respectively; total *n* = 14) and maternal lactation “not yet established” (7, 4, and 4; total *n* = 15); the corresponding estimates are imprecise and should be interpreted with caution. Maternal lactation status, maternal breastfeeding fatigue, and maternal feeding-related pain were father-reported. (total *n* = 14; 8/4/2 across the low/moderate/high groups) and “not yet established” (total *n* = 15; 7/4/4); the corresponding estimates are imprecise and should be interpreted with caution.

**Table 6:** Sensitivity analysis: three-step (R3STEP) multinomial logistic regression additionally adjusted for demographic covariates (*n* = 306).

Predictor (reference category)	Low self-efficacy group vs. Moderate self-efficacy group			High self-efficacy group vs. Moderate self-efficacy group		
	*β* (SE)	*P*	*OR* (95%*CI*)	*β* (SE)	*P*	*OR* (95%*CI*)
Breastfeeding knowledge (per one-point increase)	−0.143 (0.051)	0.005	0.87 (0.78–0.96)	0.060 (0.074)	0.419	1.06 (0.92–1.23)
Prior attendance at breastfeeding education sessions: no (ref: yes)	0.686 (0.440)	0.119	1.99 (0.84–4.70)	−0.608 (0.394)	0.123	0.54 (0.25–1.18)
Paternal perceived importance of breastfeeding: generally important (ref: very important)	0.598 (0.478)	0.211	1.82 (0.71–4.64)	−1.889 (0.445)	<0.001	0.15 (0.06–0.36)
Paternal perceived importance of breastfeeding: not important (ref: very important)	2.177 (0.964)	0.024	8.82 (1.33–58.35)	0.279 (0.879)	0.751	1.32 (0.24–7.40)
Maternal feeding-related pain: moderate-to-severe pain (ref: no or mild pain)	0.160 (0.425)	0.706	1.17 (0.51–2.70)	−0.314 (0.408)	0.441	0.73 (0.33–1.63)
Maternal breastfeeding fatigue: mild fatigue (ref: no fatigue)	1.128 (0.765)	0.140	3.09 (0.69–13.84)	−0.211 (0.546)	0.699	0.81 (0.28–2.36)
Maternal breastfeeding fatigue: moderate-to-severe fatigue (ref: no fatigue)	1.608 (0.790)	0.042	4.99 (1.06–23.49)	−0.256 (0.596)	0.668	0.77 (0.24–2.49)
Maternal lactation status: insufficient (ref: sufficient)	0.647 (0.424)	0.127	1.91 (0.83–4.38)	−0.785 (0.408)	0.054	0.46 (0.21–1.01)
Maternal lactation status: not yet established (ref: sufficient)	1.384 (0.935)	0.139	3.99 (0.64–24.94)	1.183 (1.101)	0.283	3.26 (0.38–28.25)
Feeding method before discharge: non-exclusive breastfeeding (ref: exclusive breastfeeding)	0.331 (0.414)	0.424	1.39 (0.62–3.13)	0.567 (0.401)	0.157	1.76 (0.80–3.87)
Expected feeding method: non-exclusive breastfeeding (ref: exclusive breastfeeding)	0.811 (0.423)	0.055	2.25 (0.98–5.16)	−0.907 (0.446)	0.042	0.40 (0.17–0.97)
Paternal age: ≤25 years (ref: 25–35 years)	– (not estimable)	–	–	0.657 (1.071)	0.540	1.93 (0.24–15.74)
Paternal age: >35 years (ref: 25–35 years)	0.945 (0.498)	0.058	2.57 (0.97–6.83)	1.191 (0.577)	0.039	3.29 (1.06–10.20)
Education level: master’s degree or higher (ref: undergraduate degree)	0.226 (0.440)	0.607	1.25 (0.53–2.97)	0.040 (0.469)	0.931	1.04 (0.42–2.61)
Education level: specialized degree or lower (ref: undergraduate degree)	−0.391 (0.463)	0.399	0.68 (0.27–1.68)	0.356 (0.458)	0.437	1.43 (0.58–3.50)
Household per-capita monthly income:<10,000 yuan (ref: 10,000–20,000 yuan)	0.832 (0.420)	0.048	2.30 (1.01–5.23)	0.148 (0.481)	0.759	1.16 (0.45–2.98)
Household per-capita monthly income:>20,000 yuan (ref: 10,000–20,000 yuan)	0.025 (0.472)	0.958	1.03 (0.41–2.59)	0.158 (0.461)	0.731	1.17 (0.47–2.89)

Three-step (R3STEP) estimates from Mplus 8.3 adjusted for paternal age, education level, and household per-capita monthly income. Reference outcome category: moderate self-efficacy group (*n* = 198). Model likelihood-ratio χ^2^ (34) = 132.28, *p* < 0.001; McFadden *R*^2^ = 0.242; Cox–Snell *R*^2^ = 0.351; Nagelkerke *R*^2^ = 0.422; statistics computed from the log-likelihood values of the fitted three-step models. The demographic block did not significantly improve model fit over the main model (Δχ^2^(12) = 17.67, *p* = 0.126). No father aged 25 years or younger was classified in the low self-efficacy group (0/9/2 across the low/moderate/high groups), so the corresponding coefficient is not estimable (quasi-complete separation) and is not reported. 95% CIs were computed from robust (MLR) standard errors.

The main model was statistically significant (likelihood-ratio χ^2^ (22) = 114.61, *p* < 0.001; McFadden *R*^2^ = 0.210; Cox–Snell *R*^2^ = 0.312; Nagelkerke *R*^2^ = 0.375; *n* = 306). Compared with the moderate self-efficacy group, membership in the low self-efficacy group was associated with lower breastfeeding knowledge (OR = 0.90 per one-point increase, 95% CI: 0.82–0.99, *p* = 0.024) and with perceiving breastfeeding as not important rather than very important (OR = 9.44, 95% CI: 1.65–53.91, *p* = 0.012); the associations for maternal lactation not yet established (OR = 5.19, 95% CI: 0.93–28.87, *p* = 0.060) and moderate-to-severe maternal breastfeeding fatigue (OR = 3.77, 95% CI: 0.87–16.27, *p* = 0.076) were in the same direction but did not reach statistical significance. Membership in the high rather than the moderate self-efficacy group was less likely among fathers perceiving breastfeeding as only generally important rather than very important (OR = 0.18, 95% CI: 0.08–0.42, *p* < 0.001) and among those expected non-exclusive feeding (OR = 0.38, 95% CI: 0.16–0.89, *p* = 0.026). Prior attendance at breastfeeding education sessions, feeding method before discharge, feeding-related pain, and the remaining lactation and fatigue categories were not significantly associated with profile membership (Table 5).

A sensitivity model additionally adjusting for paternal age, education level, and household per-capita monthly income (Table 6) yielded essentially unchanged estimates for the eight prespecified predictors, and the demographic block did not significantly improve model fit (Δχ^2^(12) = 17.67, *p* = 0.126). Three additional associations reached statistical significance in this model: moderate-to-severe maternal breastfeeding fatigue was associated with higher odds of membership in the low self-efficacy group (OR = 4.99, 95% CI: 1.06–23.49, *p* = 0.042); lower household income (per capita monthly income below 10,000 yuan) was likewise associated with membership in the low group (OR = 2.30, 95% CI: 1.01–5.23, *p* = 0.048); and paternal age over 35 years was associated with membership in the high self-efficacy group (OR = 3.29, 95% CI: 1.06–10.20, *p* = 0.039). Because these associations emerged only after covariate adjustment and were imprecisely estimated, they should be regarded as exploratory and warrant replication in larger samples.

## Discussion

### Current status and characteristics of paternal breastfeeding support self-efficacy in the early postpartum period

In this sample of fathers surveyed before hospital discharge in the early postpartum period, the mean FBSES-SF item score was 3.79 ± 0.71 on a 1–5 scale, descriptively comparable to values reported in previous paternal samples (19, 28). Because no validated cut-offs exist for the FBSES-SF, we refrain from categorizing the absolute level of self-efficacy. One possible contextual hypothesis is that the sample was drawn from a tertiary hospital in Shanghai, a setting with relatively high socioeconomic resources, comparatively generous parental (including paternity) leave policies, and abundant perinatal healthcare resources, however, these contextual variables were not measured and no comparison group was available, so this interpretation is speculative and should be tested in multi-regional studies. The item “I can help my spouse determine whether the baby is fully fed.” recorded the lowest average score at (3.51 ± 1.025). Previous research indicates that fathers often lack practical breastfeeding knowledge—for example, in assessing whether an infant is satiated—likely due to limited access to breastfeeding education, low participation rates, and a lack of targeted guidance (19, 36). These observations suggest that including fathers in breastfeeding education and skills training alongside mothers may be a useful direction for clinical practice, although the effectiveness of such approaches was not evaluated in the present study.

### Latent profile analysis of paternal support breastfeeding self-efficacy

Latent profile analysis identified three distinct classes of paternal breastfeeding support self-efficacy: low self-efficacy group (18.6%), moderate self-efficacy group (64.7%), and high self-efficacy group (16.7%). Notably, the three profiles were largely parallel in shape and differed primarily in overall level, suggesting that they mainly represent severity gradations of the construct; nevertheless, the profiles showed significant item-level differences (Wald χ^2^(26) = 107.983, *p* < 0.001) and differed on external variables not used in the classification, such as expected feeding method and breastfeeding knowledge, supporting their external validity. The categorical representation was retained for its clinical and communicative utility. It delineates a low self-efficacy subgroup that services may wish to prioritize, supporting their external validity. Profile membership differed across measured external variables, and the class-specific means indicate clear separation between the low (item mean 2.65 ± 0.48), moderate (3.86 ± 0.25), and high (4.80 ± 0.22) groups. Whether fathers at different self-efficacy levels benefit from different forms of support is a hypothesis that future intervention studies should test.

### Factors associated with profile membership

#### Paternal attitudes and expectations

Fathers who expected exclusive breastfeeding were more likely to belong to the “high self-efficacy group” rather than the moderate self-efficacy group (OR = 0.38, 95% CI: 0.16–0.89, *p* = 0.026 for non-exclusive versus exclusive expectations), whereas expected feeding method was not significantly associated with membership in the low self-efficacy group (*p* = 0.101). This finding is consistent with the results reported by McAlpine (37). Within Social Cognitive Theory, fathers’ preference for exclusive breastfeeding signals a stronger internalization of breastfeeding’s value. Through continued practical involvement, fathers accumulate mastery experiences that, in turn, reinforce their self-efficacy. Also, fathers with higher confidence may be more inclined to intend exclusive breastfeeding. Similarly, perceiving breastfeeding as not important was strongly associated with membership in the low versus the moderate self-efficacy group (OR = 9.44, 95% CI:1.65–53.91, *p* = 0.012), and perceiving it as only generally important (rather than very important) was associated with markedly lower odds of membership in the high group (OR = 0.18, 95% CI:0.08–0.42, *p* < 0.001), consistent with findings from Thai fathers (38, 39). These estimates, particularly the former, rest on a small subgroup (*n* = 14) and are imprecise; they should be interpreted as signals rather than definitive effect sizes.

#### Breastfeeding knowledge

Higher breastfeeding knowledge scores were associated with lower odds of membership in the low rather than the moderate self-efficacy group (OR = 0.90 per one-point increase, 95% CI: 0.82–0.99, *p* = 0.024), whereas knowledge was not significantly associated with membership in the high self-efficacy group (*p* = 0.302). This pattern, in line with results reported by Zeng (19) and Aktaş (40), suggests that foundational knowledge deficits may be particularly relevant among fathers with low self-efficacy. This may be because adequate breastfeeding knowledge enables fathers to clearly understand their roles and responsibilities, acquire practical skills (e.g., assisting with feeding and assessing satiety), and strengthen their confidence in supporting breastfeeding (19). Equally, fathers with higher self-efficacy may seek out more information, so the association should not be read as causal. Fathers are often excluded from breastfeeding education, which remains largely mother-focused (41). A systematic review of interventions targeting fathers concluded that such interventions can improve paternal breastfeeding knowledge, attitudes, and exclusive breastfeeding rates (13), supporting further development and evaluation of father-inclusive education.

Prior attendance at breastfeeding education sessions. In univariate comparisons, attendance differed significantly across profiles (26.3%, 44.4%, and 66.7% in the low, moderate, and high groups, respectively); however, in the multivariable three-step model the association was attenuated and no longer statistically significant (low vs. moderate: OR = 1.93, 95% CI: 0.88–4.23, *p* = 0.101; high vs. moderate: OR = 0.55, 95% CI: 0.26–1.17, *p* = 0.122). This attenuation after simultaneous adjustment for attitudes, knowledge, and lactation context suggests that the univariate contrast may partly reflect confounding—for example, fathers with more positive attitudes may be more likely both to attend education sessions and to report higher self-efficacy. Although previous studies have reported benefits of father-targeted education (42), the non-significant adjusted estimates should not be interpreted as evidence of no association, given the limited precision in the smaller classes; adequately powered longitudinal studies are needed.

#### Maternal lactation status

Maternal milk supply differed markedly across the three profiles in the univariate analysis (Table 1). In the multivariable three-step model, fathers whose partners had not yet established lactation tended to be more likely to belong to either the low or the high self-efficacy group than to the moderate group, but none of these associations reached statistical significance and they were imprecisely estimated (low vs. moderate: OR = 5.19, 95% CI: 0.93–28.87, *p* = 0.060; high vs. moderate: OR = 4.07, 95% CI: 0.61–27.01, *p* = 0.147); insufficient milk supply was likewise not significantly associated with profile membership after adjustment (low vs. moderate: OR = 1.84, *p* = 0.123; high vs. moderate: OR = 0.55, *p* = 0.125).

This suggests that while adequate maternal milk supply is a necessary condition for successful breastfeeding, it is not sufficient on its own—a finding consistent with the systematic review by Huang (43). This pattern may be explained by the fact that early lactation initiation is critical for breastfeeding success, and perceived insufficient supply often leads to discontinuation (44). However, even when milk production is adequate, factors such as infant irritability, frequent feeding, maternal nipple pain, and emotional distress can erode fathers’ confidence and self-efficacy (45). Thus, interventions for moderate-efficacy fathers should address not only lactation but also the practical and emotional challenges of breastfeeding support. These findings should be interpreted cautiously: the “not yet established” category was small (*n* = 15), maternal lactation variables were reported by fathers rather than assessed clinically, and the direction of any association is ambiguous—perceived lactation difficulties may erode paternal confidence, but low-confidence fathers may also be more likely to perceive feeding problems (43, 45). The attenuation of the univariate differences after adjustment suggests that lactation status may operate jointly with, or through, related factors such as feeding-related pain and fatigue rather than as an independent correlate; larger samples are needed to clarify these associations.

### Limitations

However, this study has several limitations. First, the cross-sectional design with same-time measurement of predictors and outcome precludes causal or directional inference; longitudinal cohort or intervention studies are needed to confirm the direction of these associations. Second, the sample was recruited from a single tertiary hospital in Shanghai, and selection and nonresponse bias are possible, limiting generalizability to other regions and cultural contexts. Third, all measures relied on paternal self-report and are subject to reporting and social-desirability bias; in particular, maternal lactation status, maternal breastfeeding fatigue, and maternal feeding-related pain were reported by fathers rather than by mothers or clinicians, so misclassification is possible. Fourth, residual confounding by unmeasured variables (e.g., couple relationship quality, household support structure, paternal mental health) cannot be excluded; future research could adopt a dyadic approach informed by Family Systems Theory. Fifth, cell counts were sparse for some predictor categories (perceived importance “not important,” *n* = 14; milk not yet established, *n* = 15), and the corresponding regression estimates are imprecise and vulnerable to sparse-data bias. Sixth, although the three-step R3STEP procedure accounts for classification error, latent class assignment uncertainty is reduced rather than eliminated; moreover, given the high internal consistency and the parallel shape of the profiles, the categorical representation coexists with an equally defensible continuous interpretation of the FBSES-SF, and we interpret the profiles as severity gradations. Seventh, the breastfeeding knowledge questionnaire was originally developed for mothers and, although it has been used in paternal samples with acceptable internal consistency, its full psychometric properties have not been formally validated in fathers. Finally, no validated cut-offs exist for the FBSES-SF, precluding categorical interpretation of absolute score levels.

## Conclusion

This study found that paternal breastfeeding support self-efficacy was heterogeneous and was summarized by three latent profiles: low self-efficacy group (18.6%), moderate self-efficacy group (64.7%), and high self-efficacy group (16.7%), which primarily reflect gradations of overall level. Breastfeeding knowledge, paternal perceived importance of breastfeeding, and expected feeding method were associated with profile membership; maternal breastfeeding fatigue, household per capita monthly income, and paternal age were additionally implicated in an exploratory sensitivity analysis adjusted for demographic covariates. These findings may help healthcare providers recognize fathers who could benefit from additional support and may inform the development of stratified support strategies; however, this study did not test any intervention, and whether profile-tailored strategies improve paternal support behaviors or breastfeeding outcomes remains a hypothesis for future longitudinal and interventional research. Future studies could further explore assigning fathers with high self-efficacy as peer mentors, and assess their willingness, communication ability, acceptability, and effectiveness, thereby validating this hypothesis.

## Data Availability

The original contributions presented in the study are included in the article/Supplementary material, further inquiries can be directed to the corresponding authors.

## Footnotes

1
https://www.wjx.cn

## Supplementary material

SUPPLEMENTARY Material 1 English version of the study questionnaire.

SUPPLEMENTARY Material 2 Original Chinese version of the questionnaire administered to participants.

## References

[1] World Health Organization (2023). Infant and Young Child Feeding Geneva World Health Organization. Available online at: https://www.who.int/vietnam/news/fact-sheets/detail/infant-and-young-child-feeding (accessed October 3, 2026).

[2] Smilowitz JT Allen LH Dallas DC McManaman J Raiten DJ Rozga M . Ecologies, synergies, and biological systems shaping human milk composition—a report from the “breastmilk ecology: genesis of infant nutrition (BEGIN)” working group 2. Am J Clin Nutr. (2023) 117:S28–42. doi: 10.1016/j.ajcnut.2022.11.027 PMC10356566 37173059

[3] Basree MM Shinde N Koivisto C Cuitino M Kladney R Zhang J . Abrupt involution induces inflammation, estrogenic signaling, and hyperplasia linking lack of breastfeeding with increased risk of breast cancer. Breast Cancer Res. (2019) 21:80. doi: 10.1186/s13058-019-1163-7, 31315645 PMC6637535

[4] Lubis PN Saputra M Rabbani MW. A systematic review of the benefits of breastfeeding against postpartum depression in low-middle-income countries. J Ment Health. (2025) 34:305–17. doi: 10.1080/09638237.2024.2361232, 38869015

[5] Victora CG Bahl R Barros AJ França GV Horton S Krasevec J . Breastfeeding in the 21st century: epidemiology, mechanisms, and lifelong effect. Lancet. (2016) 387:475–90. doi: 10.1016/S0140-6736(15)01024-7, 26869575

[6] Coo S García MI Prieto F. Quality of mother-infant interaction, breastfeeding, and perinatal mental health. Infant Behav Dev. (2024) 75:101946. doi: 10.1016/j.infbeh.2024.101946, 38552559

[7] Horta BL Rollins N Dias MS Garcez V Pérez-Escamilla R. Systematic review and meta-analysis of breastfeeding and later overweight or obesity expands on previous study for World Health Organization. Acta Paediatr. (2023) 112:34–41. doi: 10.1111/apa.16460, 35727183

[8] Patnode CD Henrikson NB Webber EM Blasi PR Senger CA Guirguis-Blake JM. Breastfeeding and health outcomes for infants and children: a systematic review. Pediatrics. (2025) 156:e2025071516. doi: 10.1542/peds.2025-071516, 40240318 PMC13674295

[9] Kim KM Choi JW. Associations between breastfeeding and cognitive function in children from early childhood to school age: a prospective birth cohort study. Int Breastfeed J. (2020) 15:83. doi: 10.1186/s13006-020-00326-4, 32993704 PMC7526146

[10] Oddy WH Li J Whitehouse AJ Zubrick SR Malacova E. Breastfeeding duration and academic achievement at 10 years. Pediatrics. (2011) 127:e137–45. doi: 10.1542/peds.2009-3489, 21172993

[11] Kang L Liang J He C Miao L Li X Dai L . Breastfeeding practice in China from 2013 to 2018: a study from a national dynamic follow-up surveillance. BMC Public Health. (2021) 21:329. doi: 10.1186/s12889-021-10211-2, 33568130 PMC7874457

[12] UNICEF, World Health Organization. (2024). Global Breastfeeding Scorecard 2024: Meeting the Global Target for Breastfeeding Requires Bold Commitments and Accelerated Action by Governments and Donors. New York: UNICEF. Available online at: https://clearinghouse.unicef.org/sites/ch/files/ch/sites-PD-Nutrition-Child%20Nutrition%20and%20Development%20KU-GBC-Breastfeeding-scorecard-2024-10.0.pdf (accessed October 3, 2026).

[13] Hosking T Cassidy S Louie JCY. Impact of paternal breastfeeding interventions on exclusive breastfeeding rates and attitudes of fathers towards breastfeeding: a systematic review. Curr Nutr Rep. (2025) 14:6. doi: 10.1007/s13668-024-00603-2, 39753938

[14] Leng RNW Shorey S Yin SLK Chan CPP He HG. Fathers' involvement in their wives'/partners' breastfeeding: a descriptive correlational study. J Hum Lact. (2019) 35:801–12. doi: 10.1177/0890334419830988, 30991006

[15] Rempel LA Rempel JK Moore KCJ. Relationships between types of father breastfeeding support and breastfeeding outcomes. Matern Child Nutr. (2017) 13:e12337. doi: 10.1111/mcn.12337, 27460557 PMC6865933

[16] Bandura A. Self-Efficacy: The Exercise of Control. New York: Freeman (1997).

[17] Saether KM Berg RC Fagerlund BH Glavin K Jøranson N. First-time parents' experiences related to parental self-efficacy: a scoping review. Res Nurs Health. (2023) 46:101–12. doi: 10.1002/nur.22285, 36564911 PMC10107989

[18] Hergholi M Hosseinzadeh M Sahebihagh MH Zahedi H Dahouri A. Supporting breastfeeding together: the role of fathers' attitude and self-efficacy in enhancing mothers' breastfeeding empowerment. J Health Popul Nutr. (2026) 45:45. doi: 10.1186/s41043-025-01228-z, 41645363 PMC12973900

[19] Zeng J Zheng QX Wang QS Liu GH Liu XW Lin HM . Father support breastfeeding self-efficacy positively affects exclusive breastfeeding at 6 weeks postpartum and its influencing factors in Southeast China: a multi-Centre, cross-sectional study. BMC Public Health. (2024) 24:2698. doi: 10.1186/s12889-024-20136-1, 39363194 PMC11448394

[20] Bennett AE McCartney D Kearney JM. Views of fathers in Ireland on the experience and challenges of having a breast-feeding partner. Midwifery. (2016) 40:169–76. doi: 10.1016/j.midw.2016.07.004, 27450588

[21] Brown A Davies R. Fathers' experiences of supporting breastfeeding: challenges for breastfeeding promotion and education. Matern Child Nutr. (2014) 10:510–26. doi: 10.1111/mcn.12129, 24720518 PMC4282396

[22] Chipojola R Dennis CL Kuo SY. Psychometric assessment of the paternal breastfeeding self-efficacy scale-short form: a confirmatory factor analysis of Malawian fathers. J Hum Lact. (2022) 38:28–36. doi: 10.1177/08903344211065036, 34927486

[23] Zhang W Weng Y Zhang X Shen H Li X Liu Y . Shared decision-making behavior in surgery among early breast cancer patients and associated factors using COM-B model: a latent profile analysis. J Nurs Manag. (2025) 2025:2347796. doi: 10.1155/jonm/2347796, 40703390 PMC12286671

[24] Niu Y Yang Y Li C Zhao R Hou X. Latent profile analysis of death education needs and its relationship with hospice attitude among intern nursing students. Nurse Educ Today. (2025) 153:106788. doi: 10.1016/j.nedt.2025.106788, 40411962

[25] Nylund KL Asparouhov T Muthén BO. Deciding on the number of classes in latent class analysis and growth mixture modeling: a Monte Carlo simulation study. Struct Equ Model. (2007) 14:535–69. doi: 10.1080/10705510701575396

[26] Nylund-Gibson K Choi AY. Ten frequently asked questions about latent class analysis. Transl Issues Psychol Sci. (2018) 4:440–61. doi: 10.1037/tps0000176

[27] Tein JY Coxe S Cham H. Statistical power to detect the correct number of classes in latent profile analysis. Struct Equ Modeling. (2013) 20:640–57. doi: 10.1080/10705511.2013.824781, 24489457 PMC3904803

[28] Dennis CL Brennenstuhl S Abbass-Dick J. Measuring paternal breastfeeding self-efficacy: a psychometric evaluation of the breastfeeding self-efficacy scale-short form among fathers. Midwifery. (2018) 64:17–22. doi: 10.1016/j.midw.2018.05.005, 29864577

[29] Zhou Y Xue C Jin H Zhang M Shen Z Hu H . The reliability and validity of the translated Chinese version of the father support breastfeeding self-efficacy scale-short form. Chin J Nurs. (2020) 55:1116–20. doi: 10.3761/j.issn.0254-1769.2020.07.031

[30] Zhao M. Factors Associated With Breastfeeding Self-Efficacy among Beijing New Mothers [Master's thesis]. Beijing: Peking Union Medical College (2008).

[31] Chen M Wang Y Zhang F Dai Y. Current status and influencing factors of infant fathers' support for breastfeeding. J Bengbu Med Univ. (2026) 51:256–260. doi: 10.13898/j.cnki.issn.2097-5252.2026.02.024

[32] Rhemtulla M Brosseau-Liard PE Savalei V. When can categorical variables be treated as continuous? A comparison of robust continuous and categorical SEM estimation methods under suboptimal conditions. Psychol Methods. (2012) 17:354–73. doi: 10.1037/a0029315, 22799625

[33] Bauer DJ Curran PJ. Distributional assumptions of growth mixture models: implications for overextraction of latent trajectory classes. Psychol Methods. (2003) 8:338–63. doi: 10.1037/1082-989X.8.3.338, 14596495

[34] Vermunt JK. Latent class modeling with covariates: two improved three-step approaches. Polit Anal. (2010) 18:450–69. doi: 10.1093/pan/mpq025

[35] Asparouhov T Muthén B. Auxiliary variables in mixture modeling: three-step approaches using Mplus. Struct Equ Model Multidiscip J. (2014) 21:329–41. doi: 10.1080/10705511.2014.915181

[36] Gebremariam KT Wynter K Zheng M Rawstorn JC Denney-Wilson E Laws R. Breastfeeding—a survey of fathers' support needs and preferred sources of information. Int Breastfeed J. (2024) 19:50. doi: 10.1186/s13006-024-00654-9, 39020376 PMC11256481

[37] McAlpine T Charlesworth J Martin A Scott J Mullan B. An extension of the theory of planned behaviour to predict exclusive breastfeeding among Australian mother-father dyads using structural equation modelling. Br J Health Psychol. (2025) 30:e12760. doi: 10.1111/bjhp.12760, 39435891

[38] Krikitrat P Park CG McCreary LL Koenig MD Abboud S Sansiriphun N . Relationships between Thai fathers' self-efficacy to support breastfeeding and exclusive breastfeeding duration. Midwifery. (2022) 106:103261. doi: 10.1016/j.midw.2022.103261, 35085913

[39] Krikitrat P Sansiriphun N Deeluea J Sonted S Chaiwipassatorn W Bressington D. Factors affecting Thai fathers' self-efficacy to support exclusive breastfeeding. Nurs Rep. (2023) 13:1511–23. doi: 10.3390/nursrep13040127, 37987406 PMC10660752

[40] Aktaş Reyhan F. The effect of breastfeeding education with digital storytelling on fathers' breastfeeding self-efficacy. J Eval Clin Pract. (2025) 31:e14102. doi: 10.1111/jep.14102, 39038176

[41] Pereira TL Rajendran PD Nantsupawat A Shorey S. Fathers' breastfeeding knowledge, attitudes, and involvement in the Asian context: a mixed-studies review. Midwifery. (2024) 131:103956. doi: 10.1016/j.midw.2024.103956, 38401252

[42] Koksal Karanfil I Acikgoz A Yigit D. The effect of breastfeeding education provided to fathers on breastfeeding, parent-infant bonding, and breastfeeding self-efficacy levels. J Perinat Neonatal Nurs. (2026) 40:187–98. doi: 10.1097/JPN.0000000000001003, 42080646

[43] Huang Y Liu Y Yu XY Zeng TY. The rates and factors of perceived insufficient milk supply: a systematic review. Matern Child Nutr. (2022) 18:e13255. doi: 10.1111/mcn.13255, 34382733 PMC8710095

[44] Froh EB Lee R Spatz DL. The critical window of opportunity: lactation initiation following cesarean birth. Breastfeed Med. (2021) 16:258–63. doi: 10.1089/bfm.2020.0155, 33347346

[45] Chipojola R Huda MH Gondwe KW Mbeye NM Kuo SY. Relationship and determinants of breastfeeding self-efficacy among postpartum mothers and fathers in Malawi: a cross-sectional study. J Hum Lact. (2025) 41:272–82. doi: 10.1177/08903344251318274, 40071765

