# Supplementary material for: A latent profile analysis of father support breastfeeding self-efficacy in the early postpartum period: a cross-sectional study
Source: Front Nutr. 2026 Oct 27;13:1951173. doi: 10.3389/fnut.2026.1951173 (PMC13747795; doi:10.3389/fnut.2026.1951173)
Supplement: Supplementary file 1 [file Supplementary_file_1.docx]

**Supplementary File 1. Study questionnaire**

(English translation of the Chinese questionnaire administered in the study; the translation is provided for international readers and was not used for data collection)

Dear family member of the mother,

Hello! Thank you very much for taking the time to participate in this survey. This study assesses your confidence in supporting the mother’s breastfeeding. It is conducted for research purposes only and will not have any impact on you or the mother. Completing the questionnaire takes about 5 minutes. Please answer each item truthfully according to your own situation. Your information will be kept strictly confidential. Thank you for your cooperation!

**Part A. General Information**

1. Age: ________ years

2. Contact information: ____________

3. Education level: □ Master’s degree or higher	□ Bachelor’s (undergraduate) degree	□ Specialized (junior college) degree or lower

4. Occupation: □ Company employee	□ Other work	□ Self-employed / freelance

5. Household per capita monthly household income: □ <10,000 yuan	□ 10,000–20,000 yuan	□ >20,000 yuan

6. Household registration: □ Shanghai (local)	□ Other regions

7. Mother’s name: ____________	Mother’s age: ________

8. Mode of delivery:

□ Vaginal delivery(include operative vaginal deliveries)	□ Cesarean section

9. Were there any complications or comorbidities during this pregnancy (e.g., hypertension, diabetes, heart disease, hyperthyroidism or hypothyroidism)?

□ No	□ Yes, please specify: ____________

10. Parity: □ First birth	□ Second birth	□ Third birth or more

11. Neonate: ① Sex: □ Boy □ Girl	② Birth weight: ________ g

12. Which feeding method is your partner currently using (before discharge)?

□ Exclusive breastfeeding (breast milk only, nothing else)

□ Mixed feeding (breast milk supplemented with formula or other milk substitutes)

□ Artificial feeding (formula or other milk substitutes only)

13. Which feeding method do you expect to use after discharge?

□ Exclusive breastfeeding (breast milk only, nothing else)

□ Mixed feeding (breast milk supplemented with formula or other milk substitutes)

□ Artificial feeding (formula or other milk substitutes only)

14. Do you think breastfeeding is important?

□ Not important at all	□ Not very important	□ Generally important	□ Important	□ Very important

15. Have you previously attended breastfeeding education courses together with your partner?

□ Yes	□ No

16. How is your partner’s current milk supply?

□ Sufficient milk	□ Insufficient milk	□ Not yet established

17. How is your partner’s current fatigue with breastfeeding?

□ No fatigue	□ Mild fatigue	□ Moderate fatigue	□ Severe fatigue

18. How much nipple pain does your partner have during breastfeeding?

□ No pain	□ Mild pain	□ Moderate pain	□ Severe pain

**Part B. Father Support Breastfeeding Self-Efficacy Scale–Short Form (FBSES-SF), Chinese Version**

Please answer according to your family’s actual situation and mark "√" in the box of the option you consider correct. Choose only one option for each item.

| Item | Not at all confident | Not very confident | Sometimes confident | Confident | Very confident |
| --- | --- | --- | --- | --- | --- |
| 1. I can help my partner ensure that the baby has enough breast milk. |  |  |  |  |  |
| 2. I can help my partner with breastfeeding, just as I would help her deal with other challenging tasks. |  |  |  |  |  |
| 3. I can help my partner breastfeed the baby without using formula. |  |  |  |  |  |
| 4. I can help my partner ensure that the baby latches onto the nipple correctly and suckles throughout the feeding. |  |  |  |  |  |
| 5. I can help my partner manage our breastfeeding situation to our satisfaction. |  |  |  |  |  |
| 6. Even when our baby cries, I can help my partner continue breastfeeding. |  |  |  |  |  |
| 7. I can help my partner maintain her willingness to continue breastfeeding. |  |  |  |  |  |
| 8. I can help my partner feel relaxed and not embarrassed while breastfeeding, even when family members are present. |  |  |  |  |  |
| 9. I am satisfied with our breastfeeding situation. |  |  |  |  |  |
| 10. Although breastfeeding is time-consuming, I can help my partner cope with it. |  |  |  |  |  |
| 11. I can help my partner finish feeding on one breast before switching to the other. |  |  |  |  |  |
| 12. I can help my partner ensure that each feeding is completed without interruption. |  |  |  |  |  |
| 13. I can help my partner feed the baby according to the baby’s demand for breast milk. |  |  |  |  |  |
| 14. I can help my partner determine whether the baby is fully fed. |  |  |  |  |  |

Note: The FBSES-SF was developed by Dennis et al. (2018) to assess fathers’ confidence in supporting breastfeeding; higher scores indicate greater self-efficacy. The 14 items are rated on a 5-point Likert scale (total score 14–70). The Chinese version was localized and validated by Zhou et al. (2020).

# **Part C. Breastfeeding Knowledge Questionnaire**

Please answer according to your family’s actual situation and mark "√" in the box of the option you consider correct. Choose only one option for each item.

| Item | Yes | No | Uncertain |
| --- | --- | --- | --- |
| 1. Newborns should be fed on a fixed schedule rather than on demand (demand feeding means feeding whenever the infant is hungry or the mother’s breasts are engorged, without restrictions on feeding frequency or interval). |  |  |  |
| 2. The earlier breastfeeding is initiated after childbirth, the better. |  |  |  |
| 3. The amount of milk secreted is related to how often the infant suckles, i.e., "the more the infant suckles, the more milk is produced". |  |  |  |
| 4. Breastfeeding can prevent childhood obesity. |  |  |  |
| 5. Breastfeeding can prevent infantile rickets (a disease characterized by bone lesions caused by calcium deficiency). |  |  |  |
| 6. Breastfeeding can prevent infant diarrhea. |  |  |  |
| 7. Breastfeeding can promote infant intellectual development. |  |  |  |
| 8. Breastfeeding can reduce the occurrence of maternal breast diseases. |  |  |  |
| 9. Breastfeeding can reduce the occurrence of maternal breast cancer. |  |  |  |
| 10. Breastfeeding can reduce postpartum hemorrhage and promote maternal postpartum recovery. |  |  |  |
| 11. Breastfeeding can enhance mother–infant bonding. |  |  |  |
| 12. Breast milk can fully meet all the nutritional needs of an infant under six months of age, without any additional food or drink. |  |  |  |
| 13. Remaining milk in the breasts should not be expressed after a feeding. |  |  |  |
| 14. For infants, the nutritional value of breast milk is greater than that of fresh cow’s milk or formula. |  |  |  |
| 15. Exclusive breastfeeding should be continued for at least four months after childbirth. |  |  |  |
| 16. When breastfeeding, the infant’s chin should touch the breast, and the nipple and most of the areola should be taken into the infant’s mouth. |  |  |  |
| 17. The infant’s daily urine output is a reliable indicator of whether the infant is receiving enough breast milk. |  |  |  |

Note: The 17-item Breastfeeding Knowledge Questionnaire was originally developed by Zhao (2008) and covers two domains: the benefits of breastfeeding and breastfeeding skills. Each correct response is scored 1 point (incorrect or uncertain, 0); the total score ranges from 0 to 17, with higher scores indicating more comprehensive breastfeeding knowledge. For the present study, item wording was minimally adapted so that all items were answerable by fathers.
