# Supplementary material for: A latent profile analysis of father support breastfeeding self-efficacy in the early postpartum period: a cross-sectional study
Source: Front Nutr. 2026 Oct 27;13:1951173. doi: 10.3389/fnut.2026.1951173 (PMC13747795; doi:10.3389/fnut.2026.1951173)
Supplement: Supplementary file 2 [file Supplementary_file_2.docx]

**父亲支持母乳喂养自我效能的影响因素调查问卷**

亲爱的产妇家属：

您好！首先感谢您能抽出时间参加本次调研，本研究是评估您对支持产妇母乳喂养的信心程度，仅做研究不会对您和产妇造成任何影响，问卷填写仅需5分钟左右，请您根据自身情况如实填写问卷，我们会对您的信息保密，谢谢合作！

**一般资料**

1.年龄：	岁

2.联系方式：____________

3.学历：□硕士及以上 □本科 □大专及以下

4.职业：□公司职员 □其他工作 □自由职业

5.家庭人均月收入：□＜10000元 □10000-20000元 □＞20000元

6.户籍：□上海本地 □外地

7.产妇姓名：	年龄__________

8.分娩方式：□顺产 □剖腹产

9.此次妊娠有无并发症或合并症（如高血压、糖尿病、心脏病、甲亢或甲减等）：

□无	□有	何种并发症__________

10.胎次：□一胎 □二胎 □三胎

11.新生儿性别：①□男 □女	②体重：	g

12.您目前（出院前）是采用哪种喂养方式？

□纯母乳喂养（只喂母乳，不加任何东西）□混合喂养（母乳喂养之外补充其他代乳品）□人工喂养（完全由代乳品等喂养

13.您期望出院后将采用何种喂养方式？

□纯母乳喂养（只喂母乳，不加任何东西）□混合喂养（母乳喂养之外补充其他代乳品）□人工喂养（完全由代乳品等喂养）

14.您认为母乳喂养重要吗？

□非常不重要 □不太重要 □较为重要 □重要 □非常重要

15.您既往是否与伴侣共同参加过母乳喂养教育课程？

□是 □否

16.您伴侣当前乳汁分泌情况如何？

□乳汁充足 □乳汁不足 □尚无乳汁

17.您伴侣母乳喂养疲劳情况如何？

□无疲劳 □轻度疲劳 □中度疲劳 □重度疲劳

18.您伴侣母乳喂养乳头疼痛情况如何？

□无疼痛	□轻度疼痛 □中度疼痛 □重度疼痛

**中文版父亲支持母乳喂养自我效能（FBSES-SF）量表**

请根据您的实际情况作答，在您认为正确的选项空格内划“√”，每题只选择一项。

| 项目 | 一点也没有信心 | 不是很有信心 | 有时有信心 | 有信心 | 非常有信心 |
| --- | --- | --- | --- | --- | --- |
| 1.我能帮助妈妈确保宝宝母乳充足 |  |  |  |  |  |
| 2.我能帮助妈妈做好母乳喂养，就像帮助她应付其他有挑战的事情一样 |  |  |  |  |  |
| 3.我能帮助妈妈在不使用配方奶粉的情况下母乳喂养宝宝 |  |  |  |  |  |
| 4.我能帮助妈妈确保宝宝在整个吃奶过程中正确含住乳头进行吸吮 |  |  |  |  |  |
| 5.我能帮助妈妈将母乳喂养的状况控制到令我们满意 |  |  |  |  |  |
| 6.即使我们的宝宝哭泣，我也能帮助妈妈将哺乳进行下去 |  |  |  |  |  |
| 7.我能让妈妈坚持母乳喂养的意愿 |  |  |  |  |  |
| 8.哺乳时即使有家人在场，我也能帮助妈妈心情放松而不会感到尴尬 |  |  |  |  |  |
| 9.我对我们母乳喂养的状况感到满意 |  |  |  |  |  |
| 10.虽然母乳喂养比较耗时，我也能帮助妈妈应付 |  |  |  |  |  |
| 11.我能帮助妈妈把一边乳房喂完了再换另一个 |  |  |  |  |  |
| 12. 我能帮助妈妈确保每次喂奶都不会间断 |  |  |  |  |  |
| 13.我能够帮助妈妈依据宝宝对母乳的需求来喂奶 |  |  |  |  |  |
| 14.我能帮助妈妈判断宝宝是否吃饱了 |  |  |  |  |  |

**母乳喂养知识问卷**

请您根据您家庭的实际情况做出回答，在您认为正确的选项空格内划“√”，每题只选择一项。

| 项目 | 是 | 否 | 不确定 |
| --- | --- | --- | --- |
| 1.新生儿应该按时喂养，而不是按需喂养（按需哺乳是支持当婴儿饿了或母亲乳房胀了就进行哺喂，哺喂的次数和间隔时间不受限制） |  |  |  |
| 2.产后开始不喂母乳的时间越早越好 |  |  |  |
| 3.乳汁分泌的多少与婴儿的吸吮次数有关，即“吸得越多，分泌越多” |  |  |  |
| 4.母乳喂养能预防儿童肥胖 |  |  |  |
| 5.母乳喂养能预防婴儿佝偻病（由于钙缺乏导致的一种以骨骼病变为特征的疾病） |  |  |  |
| 6.母乳喂养能预防婴儿腹泻 |  |  |  |
| 7.母乳喂养能促进婴儿智力发育 |  |  |  |
| 8.母乳喂养能减少母亲乳腺疾病的发生 |  |  |  |
| 9.母乳喂养能减少母亲乳腺癌的发生 |  |  |  |
| 10.母乳喂养能减少产后出血，促进母亲产后身体恢复 |  |  |  |
| 11.母乳喂养能增进母婴感情 |  |  |  |
| 12.母乳能完全满足6个月婴儿全部应用需求，而不必加任何食物和饮料 |  |  |  |
| 13.哺乳后不应该将乳房中剩余的乳汁排出 |  |  |  |
| 14.对婴儿而言，母乳的营养成分大于鲜牛奶/奶粉 |  |  |  |
| 15.纯母乳喂养应至少持续到产后4个月 |  |  |  |
| 16.在喂哺婴儿母乳时，应使婴儿的下颌贴到乳房，乳头及大部分乳晕都含进婴儿嘴里 |  |  |  |
| 17.婴儿每天尿量是判断婴儿是否得到足够母乳的可靠指标 |  |  |  |
